# School environment and oral health promotion: the National Survey of School Health (PeNSE)

**DOI:** 10.11606/s1518-8787.2019053001376

**Published:** 2019-10-16

**Authors:** Newillames Gonçalves Nery, Lidia Moraes Ribeiro Jordão, Maria do Carmo Matias Freire

**Affiliations:** I Secretaria Municipal de Saúde de Goiânia. Goiânia, GO, Brasil; II Universidade Federal de Goiás. Faculdade de Odontologia. Programa de Pós-Graduação em Odontologia. Goiânia, GO, Brasil

**Keywords:** Adolescent Health, School Dentistry Services, School Health Services, Health Disparities Oral Health, Health Promotion, Epidemiological Surveys

## Abstract

**OBJECTIVE:**

To evaluate the potential support of schools for oral health promotion and associated factors in Brazilian capitals.

**METHODS:**

Data from 1,339 public and private schools of the 27 Brazilian capitals were obtained from the National Survey of School Health (PeNSE) 2015. Data from the capitals were obtained from the United Nations Development Program and the Department of Informatics of the Brazilian Unified Health System (Datasus). The indicator “ *ambiente escolar promotor de saúde bucal* ” (AEPSB – oral health promoting school environment) was designed from 21 variables of the school environment with possible influence on students’ oral health employing the categorical principal components analysis (CATPCA). Associations between the AEPSB and characteristics of schools, capitals and regions were tested (bivariate analysis).

**RESULTS:**

Ten variables comprised CAPTCA, after excluding those with low correlation or high multicollinearity. The analysis resulted in a model with three dimensions: D1. Within-school aspects (sales of food with added sugar in the canteen and health promotion actions), D2. Aspects of the area around the school (sales of food with added sugar in alternative points) and D3. prohibitive policies at school (prohibition of alcohol and tobacco consumption). The sum of the scores of the dimensions generated the AEPSB indicator, dichotomized by the median. From the total of schools studied, 51.2% (95%CI 48.5–53.8) presented a more favorable environment for oral health (higher AEPSB). In the capitals, this percentage ranged from 36.6% (95%CI 23.4–52.2) in Rio Branco to 80.4% (95%CI 67.2–89.1) in Florianópolis. Among the Brazilian regions, it ranged from 45.5% (95%CI 40.0–51.2) in the North to 67.6% (95%CI 59.4–74.9) in the South. Higher percentages of schools with higher AEPSB were found in public schools [58.1% (95%CI 54.9–61.2)] and in capitals and regions with higher Human Development Index [61.0% (95%IC 55.8–66.0) and 57.4% (95%CI 53.2–61.4), respectively] and lower Gini index [55.7% (95%CI 51.2–60.0) and 52.8 (95%CI 49.8–55.8), respectively].

**CONCLUSIONS:**

The potential to support oral health promotion in schools in Brazilian capitals, assessed by the AEPSB indicator, was associated with contextual factors of schools, capitals and Brazilian regions.

## INTRODUCTION

The environments where people live are important components of health promotion^1^ . Environmental factors, besides the individual ones, can influence the relationship between education and health^2^ . The school is one of the strategic spaces for stimulation and development of healthier skills, behaviors and lifestyles, particularly among children and adolescents^2 , 3^ . In this sense, studies have investigated environmental factors – physical or social – and health-promoting interventions conducted at schools, and their association with conditions, perceptions and behaviors related to students’ health^4^ .

In the 1990s, the World Health Organization, recognizing this aspect in the intrinsic relationship between education and health, suggested an approach called “health promoting schools” (HPS), based on school environments considered “healthy”^7^ . In these environments, institutional health policies, school curriculum, social and physical environment, and interaction with the community, coupled with the individuals’ self-esteem and health-inducing practices, can provide support to health promotion^7^ .

Review studies on the effectiveness of the HPS approach, as well as other models for health promotion at school, show that the results of the actions are variable and limited, but with potential positive effects on individual and collective health, as well as in academic performance^4^ . Health promotion programs in the school environment with longer duration and greater involvement of the school community, as well as those addressing mental health, healthy eating and physical activities, present greater evidence of positive effects^12^ . Additionally, the availability of healthier food options within and around schools, as well as the restriction on the use of tobacco and alcohol, positively interfere with the establishment of preventive habits of non-communicable chronic diseases^13^

The relationship between school environment and oral health has also been investigated, but with fewer studies^14^ In Brazil, school environments favorable to health promotion were associated with better oral health conditions, with lower prevalence of caries and dental trauma^8 , 9 , 14^ , in addition to a better quality of life related to oral health^15^ . These associations have also been studied in other countries. In Canada, a study found an association between a more unfavorable school socioeconomic environment and a higher prevalence of pain and dental caries in public schools^16^ , besides the association between school environments with potential for oral health promotion and lower incidence of caries^17^ . In Thailand, the availability of healthy foods in the schools was associated with lower consumption of sweets and lower caries indices^18^ . A recent systematic review of worldwide studies evaluating health promotion actions and prevention of risk factors in the HPS context, showed a lack of specific activities in the school environment focused on oral health promotion^19^ .

The need for consolidated instruments to evaluate actions related to oral health promotion not only in the school environment but also in other scenarios, constitutes an important methodological and strategic challenge to be overcome^20^ . Building indicators with data from national surveys of school health may constitute an appropriate alternative to contribute to evaluation processes. The Brazilian National Survey of School Health (PeNSE), conducted triennially since 2009 by the Ministry of Health and the Brazilian Institute of Geography and Statistics (IBGE), aims to contribute to the monitoring of the health condition of Brazilian adolescent students. This survey covers several issues related to health and individual behaviors, besides variables referring to the school environment, which may influence students’ general and oral health^21^ .

Using the 2015 PeNSE data, Horta et al.^22^ elaborated the *escore de promoção de saúde no ambiente escolar* (EPSAE – health promoting school environment score), which identified school environments with better health promotion conditions. Although many risk factors for oral diseases and disorders are common for other chronic diseases^23^ , the characteristics of schools that are more directly related to oral health promotion must be considered to enable the planning and evaluation of specific actions.

Therefore, this study aimed to evaluate the potential to support oral health promotion in the school environment of Brazilian capitals and associated factors.

## METHODS

This is a cross-sectional study using the PeNSE 2015 database, available on the IBGE website^24^ . PeNSE 2015 used a complex sampling plan (by conglomerates), involving approximately 102,072 adolescent students in the ninth grade of elementary school from 3,040 public and private schools in the 27 Brazilian capitals and inner cities. The schools were selected according to information from the 2013 School Census, which constituted the most up-to-date registration at the time of the research planning. The presence of public (federal, state and municipal) and private schools in the sample was ensured in a proportion approximately similar to that existing in the selection register.

For this study, only data referring to 1,339 public and private schools of the 26 Brazilian state capitals and the Federal District were used. The data collection instrument was an electronic structured questionnaire, applied through interviews with the principals or other people in charge of the institutions. The project of PeNSE 2015 was approved by the *Conselho Nacional de Ética em Pesquisa* (CONEP – National Committee of Research Ethics), report no. 1.006.467/2015). More information on the methodological aspects of this survey can be obtained in a previous publication^21^ .

Initially, the indicator “ *ambiente escolar promotor de saúde bucal* ” (AEPSB – oral health promoting school environment) was designed to measure the potential support of the school environment for oral health promotion. In the first stage, three professionals with knowledge and experience in dental public health selected, by consensus, 21 questions from the PeNSE 2015 questionnaire with potential to influence conditions or behaviors related to adolescents’ oral health^21^ . The selected variables were: “sale of soft drinks in the canteen”, “sale of other beverages with added sugar in the canteen”, “sale of sweets and other delicacies in the canteen”, “sale of fresh fruit or fruit salads in the canteen”, “sale of soft drinks at alternative points”, “sale of other beverages with added sugar at alternative points”, “sale of sweets and other delicacies at alternative points”, “sale of fresh fruit or fruit salads at alternative points”, “school has vegetable garden”, “school has sinks in working condition”, “school has health group or committee”, “school joined the *Programa Saúde na Escola* (PSE – Health in School Program)”, “school develops actions of the PSE”, “school develops actions of the *Programa Mais Educação (* PME – More Education Program)”, “school develops actions along with the Primary Health Units (PHU)”, “school has record on students’ health data”, “school has first aid devices and/or medications”, “school is aware of teachers who smoke in the school”, “school is aware of students who smoke in the school”, “school prohibits tobacco consumption” and “school prohibits alcohol consumption”.

All these variables were initially categorized as yes or no, except for “school has sink in working condition” (yes, no, it is not in working condition) and “school has first aid devices and/or medications” (yes, no, it is not in a proper place). For this study, both were categorized as yes or no as follows: the category “it is not in working condition” was considered “no” for the first variable, and the category “it is not in a proper place” was considered “yes” for the second variable.

After analyzing the selected variables in a correlation matrix, 11 were excluded, eliminating those with low correlation (if all correlation values are < 0.4) or high multicollinearity (if any correlation value is > 0.9). Therefore, ten variables comprised the final analysis: “sale of soft drinks in the canteen”, “sale of other beverages with added sugar in the canteen”, “sale of sweets and other delicacies in the canteen”, “sale of soft drinks at alternative points”, “sale of other beverages with added sugar at alternative points”, “sale of sweets and other delicacies at alternative points”, “school develops actions of the PSE”, “school develops actions along with the PHU”, “school prohibits tobacco consumption” and “school prohibits alcohol consumption”.

Then, these ten variables were reduced to construct an indicator that summarized them. Categorical or non-linear principal component analysis (CATPCA) was used. This analysis presents results comparable to principal component analysis (PCA), which is generally used for numerical variables^25^ . This method is used to reduce categorical variables (nominal or ordinal) with the aim of decreasing data dimensionality, summarizing several variables into some non-correlated components (dimensions) with the least information loss possible. The categories of the variables are given numerical values through a process called quantification, scale or optimum score. With these numerical values, the variance of the variables is calculated^25^ .

Therefore, values are generated indicating: the variation calculated by component or dimension; the load of components (reflecting the correlations between quantified variables and principal components); the sums of the loads (eigenvalues), which reveal the contributions of variables to the total data variance (percentage of explanation) and the scores for each component, which may be useful in other analytical steps. The data synthesis presents the Cronbach’s alpha coefficient, which quantifies, in a scale from zero to one, the reliability and internal consistency of the data obtained in a questionnaire or scale, being commonly desirable a value above 0.7^25^ . In this analysis, the uninformed, lost or missing values were imputed with the mode of the quantified variable. The statistical software SPSS (version 23, SPSS Inc., Chicago, IL, USA) was used. In the final step of the CATPCA, the scores were summed up and, due to the lack of a reference value and the non-normal data distribution, the resulting variable was dichotomized based on its median, generating the AEPSB indicator.

In the next stage, descriptive and bivariate analyses were performed (Chi-square tests), seeking to identify the characteristics of schools, capitals and regions associated with the oral health promotion indicator (AEPSB). School variables were their geographic and organizational characteristics: location of the school in the capital city (rural or urban), administrative dependence (public or private), and full-time school (no or yes). The quantitative variables related to the capitals were categorized based on the terciles: the human development index (HDI) (low, medium or high) and Gini index (high, medium or low). For the regions, the parameters were: HDI (low or high) and Gini index (high or low). Statistical significance level was set at 5%.

The HDI is calculated using indicators of education, longevity and income of the population, ranging from zero to one. The closer to one, the higher the human population development of the municipality and the region^26^ . The Gini index assesses the inequality in income distribution and also varies from zero to one, but with different interpretation: the closer to one, the higher the economic inequality in the population^27^ . Data related to HDI were obtained from the database of the Human Development Atlas in Brazil^28^ , whereas Gini index values were collected from the Datasus institutional database^29^ . Both refer to 2010, which is the year with available data closest to PeNSE 2015.

## RESULTS

Table 1 shows the distribution of schools according to geographic location by regions, capital cities and organizational characteristics. Most of them were located in urban areas, were public and not full-time. Table 2 shows the final correlation matrix, including the ten variables that were analyzed in the CATPCA to generate the AEPSB indicator.

**Table 1:** Distribution of schools according to geographic location by regions and capital cities and organizational characteristics, based on the National Survey of School Health (PeNSE) 2015.

Geographic location	n	%
Regions and capital cities (States)		

North region	303	22.6
Porto Velho (RO)	46	3.4
Rio Branco (AC)	41	3.1
Manaus (AM)	35	2.6
Boa Vista (RR)	44	3.3
Belém (PA)	46	3.4
Macapá (AP)	51	3.8
Palmas (TO)	40	3.0
Northeast region	480	35.9
São Luís (MA)	54	4.0
Teresina (PI)	58	4.3
Fortaleza (CE)	46	3.4
Natal (RN)	59	4.4
João Pessoa (PB)	68	5.1
Recife (PE)	56	4.2
Maceió (AL)	41	3.1
Aracaju (SE)	54	4.0
Salvador (BA)	44	3.3
Southeast region	218	16.3
Belo Horizonte (MG)	62	4.6
Vitória (ES)	62	4.6
Rio de Janeiro (RJ)	51	3.8
São Paulo (SP)	43	3.2
South region	139	10.4
Curitiba (PR)	42	3.1
Florianópolis (SC)	51	3.8
Porto Alegre (RS)	46	3.4
Midwest region	199	14.9
Campo Grande (MS)	44	3.3
Cuiabá (MT)	39	2.9
Goiânia (GO)	68	5.1
Federal District (FD)	48	3.6

Total	1,339	100.0

Organizational characteristics		

Location in the municipality		
Rural	48	3.6
Urban	1,291	96.4
Total	1,339	100.0
Administrative dependence		
Public	940	70.2
Private	399	29.8
Total	1,339	100.0
Full-time school		
Yes	275	20.5
No	1,063	79.4

Total	1,338	100.0

**Table 2:** Matrix of final correlations with the ten variables related to the oral health promoting school environment selected for the categorical principal components analysis (CATPCA), based on the National Survey of School Health (PeNSE) 2015.

Variables		Sale of soft drinks in the canteen	Sale of other beverages with added sugar in the canteen	Sale of sweets and other delicacies in the canteen	Sale of soft drinks at alternative points	Sale of other beverages with added sugar at alternative points	Sale of sweets and other delicacies at alternative points	School develops actions of the PSE	School develops actions along with the PHU	School prohibits tobacco consumption	School prohibits alcohol consumption
Sale of soft drinks in the canteen	Pearson Correlation	1									
	p										
	n	1,304									
Sale of other beverages with added sugar in the canteen	Pearson Correlation	**0.50**	1								
	p	**< 0.001**									
	n	1,304	1,304								
Sale of sweets and other delicacies in the canteen	Pearson Correlation	**0.60**	**0.47**								
	p	**< 0.001**	**< 0.001**								
	n	1,304	1,304	1,304							
Sale of soft drinks at alternative points	Pearson Correlation	-0.02	0.02	-0.01	1						
	p	0.433	0.509	0.778							
	n	1,300	1,300	1,300	1,332						
Sale of other beverages with added sugar at alternative points	Pearson Correlation	-0.00	**0.08**	**0.06**	**0.63**	1					
	p	0.857	**0.005**	**0.023**	**< 0.001**						
	n	1,300	1,300	1,300	1,332	1,332					
Sale of sweets and other delicacies at alternative points	Pearson Correlation	0.00	0.04	**0.11**	**0.53**	**0.46**	1				
	p	0.926	0.121	**< 0.001**	**< 0.001**	**< 0.001**					
	n	1,300	1,300	1,300	1,332	1,332	1,332				
School develops actions of the PSE	Pearson Correlation	**0.27**	**0.23**	**0.22**	0.03	**0.07**	0.03	1			
	p	**< 0.001**	**< 0.001**	**< 0.001**	0.298	**0.013**	0.271				
	n	1,303	1,303	1,303	1,331	1,331	1,331	1,336			
School develops actions along with the PHU	Pearson Correlation	**0.25**	**0.22**	**0.18**	0.004	0.035	-0.026	**0.40**	1		
	p	**< 0.001**	**< 0.001**	**< 0.001**	0.873	0.200	0.350	**< 0.001**			
	n	1,303	1,303	1,303	1,331	1,331	1,331	1,335	1,336		
School prohibits tobacco consumption	Pearson Correlation	0.02	-0.01	0.01	-0.01	**-0.05**	-0.03	**0.05**	**0.07**	1	
	p	0.563	0.617	0.632	0.623	**0.050**	0.303	**0.047**	**0.013**		
	n	1,303	1,303	1,303	1,331	1,331	1,331	1,335	1,335	1,336	
School prohibits alcohol consumption	Pearson Correlation	0.01	-0.04	-0.01	-0.04	**-0.06**	**-0.07**	0.032	0.032	**0.60**	1
	p	0.684	0.149	0.755	0.141	**0.023**	**0.017**	0.240	0.238	**0.000**	
	n	1,303	1,303	1,303	1,331	1,331	1,331	1,335	1,335	1,336	1,336

p: Bilateral significance; PSE: *Programa Saúde na Escola* (Health in School Program); PHU: Primary Health Unit

Table 3 shows the results regarding the development of the AEPSB indicator. CATPCA resulted in a model with three dimensions, with an acceptable percentage of explanation of the data variance (61.2%) and high Cronbach’s alpha coefficient (0.93).

**Table 3:** Results of categorical principal components analysis (CATPCA) for the variables related to the oral health promoting school environment, based on the National Survey of School (PeNSE) 2015.

Resulting variables and dimensions	n	%	CATPCA				

			Calculated variance	Factor loadings	Eigenvalue	Percentage of variance explanation	Cronbach’s Alpha
Dimension 1 – Within-school aspects							

Canteen: soft drinks			0.566	0.752	2.419	24.2%	0.652
Yes	404	30.2					
**No**	900	67.2					
Canteen: sweets or delicacies			0.546	0.739			
Yes	324	24.2					
**No**	980	73.2					
Canteen: beverages with added sugar			0.499	0.706			
Yes	284	30.02					
**No**	1,020	76.2					
School: PSE actions			0.302	0.549			
No	796	59.4					
**Yes**	540	40.3					
School: actions with PHU			0.250	0.500			
No	840	62.7					
**Yes**	496	37.0					

Dimension 2 – Aspects of the area around the school							

Alternative sale point: soft drinks			0.656	0.810	2.099	21.0%	0.582
Yes	276	20.6					
**No**	1,056	78.9					
Alternative sale point: other beverages with added sugar			0.595	0.771			
Yes	162	12.1					
**No**	1,170	87.4					
Alternate sale point: sweets or delicacies			0.517	0.719			
Yes	244	18.2					
**No**	1,088	81.3					

Dimension 3 – Prohibitive policies							

School: prohibits tobacco consumption			0.744	0.863	1.602	16.0%	0.417
No	1,214	90.7					
**Yes**	111	8.3					
School: prohibits alcohol consumption			0.720	0.849			
No	1,225	91.5					
**Yes**	111	8.3					

Total			-	-	6.120	61.2%	0.930

PSE: *Programa Saúde na Escola* (Health in School Program); PHU: Primary Health Unit

Notes: Environmental characteristics that are potentially favorable to oral health are highlighted in bold.

The values related to the category “not informed” of each variable were considered in the analyses, but omitted in the table due to the low relative percentage (between 0.2 and 2.6%).

The percentage of explanation of variance is calculated from the quotient between the self-value ( *eigenvalue* ) and the total number of variables (in this case, ten).

The three dimensions generated were: dimension 1. within-school aspects – sales of food with added sugar in the canteen (soft drinks, other beverages with added sugar, sweets and other delicacies) and health promotion actions conducted at school (PSE actions or actions along with the PHU); dimension 2. aspects of the areas around the school – sales of foods with added sugar in alternative points near the school (soft drinks, other beverages with added sugar, sweets and other delicacies); and dimension 3. prohibitive policies – internal prohibition of alcohol and tobacco consumption in the school ( Table 3 ).

For each dimension, the CATPCA generated a score for the school environment. For dimension 1, the score ranged from -2.85 to 1.43, with a median 0.25; For dimension 2, it ranged from -3.86 to 1.96, with median 0.22; for dimension 3, it ranged from -4.57 to 1.12, with median 0.34. Scores of the three dimensions were summed up, resulting in a total score ranging from -7.46 to 2.07, with a median value of 0.64. This total score was dichotomized by the median, generating the general indicator of the potential for oral health promotion, whose categories were: higher potential (school with overall score above the median, i.e., higher AEPSB – from 0.64 to 2.07) and lower potential (school with overall score below the median, i.e., lower AEPSB – from -7.46 to 0.64).

**Figure 1:**
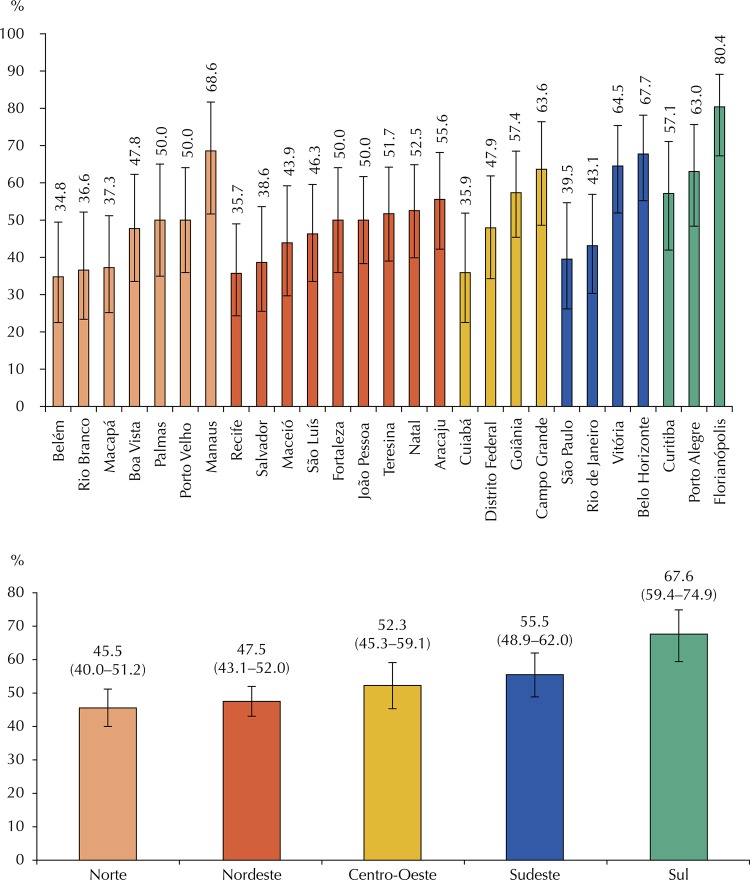
Distribution of the proportion of schools with higher potential of oral health promotion (AEPSB indicator) by capital cities and regions, based on the National Survey of School Health (PeNSE) 2015.

Note: Pearson’s Chi-square test (p < 0.001)

Using the AEPSB indicator, 685 schools (51.2%; 95%CI 48.5–53.8) had a school environment with higher potential for oral health promotion. The frequencies in each of the 27 capitals and of the five Brazilian regions are shown in Figure 1 , presenting great variations. The North and Northeast regions had lower proportions of school environments with higher AEPSB, whereas the South region had with the highest percentages (p < 0.001). Among the capitals, Florianópolis (80.39%; 95%CI 67.24–89.12) and Belo Horizonte (67.7%; 95%CI 55.2–78.2) had higher percentages of schools with higher AEPSB, whereas Belém (34.8%; 95%CI 22.5–49.5) and Recife (35.7%; 95%CI 24.3–49.0) had the lowest percentages (p < 0.001).

Significant associations were found between the potential of oral health promotion and the contextual variables related to the capitals and regions ( Table 4 ): schools with higher AEPSB were more frequent in the capitals and regions with higher HDI (p < 0.001) and lower Gini index (p < 0.05). Schools with higher AEPSB were more frequent in the public (58.1%; 95%CI 55.0–61.2) than in the private sector (34.8%; 95%CI 30.3–39.7) (p < 0.001).

**Table 4:** Distribution of the schools according to the indicator AEPSB – oral health promoting school environment ) and characteristics of the schools and capital cities, based on the National Survey of School Health (PeNSE) 2015.

Level	Schools with lower AEPSB (n = 654)			Schools with higher AEPSB (n = 685)			p*

Variables							
		
Categories	n	%	95%CI	n	%	95%CI	
School							
School location							
Rural	22	45.8	32.4–59.9	26	54.2	40.1–67.6	0.671
Urban	632	48.9	46.2–51.7	659	51.1	48.3–53.8	
Administrative dependence							
Public	394	41.9	38.8–45.1	546	58.1	54.9–61.2	< 0.001
Private	260	65.2	60.4–69.7	139	34.8	30.3–39.7	
Full-time							
No	525	49.4	46.4–52.4	538	50.6	47.6–53.6	0.401
Yes	128	46.5	40.7–52.5	147	53.5	47.5–59.3	
Capital							
Human development index							
Low	246	54.4	49.8–59.0	206	45.6	41.0–50.2	
Medium	270	50.7	46.4–54.9	263	49.3	45.1–53.6	< 0.001
High	138	39.0	34.0–44.1	216	61.0	55.8–66.0	
Gini Index							
High	201	54.0	48.9–59.0	171	46.0	41.0–51.0	
Medium	237	49.4	44.9–53.9	243	50.6	46.2–55.1	0.018
Low	216	44.4	40.0–48.8	271	55.7	51.2–60.0	
Region							
Human development index							
Low	417	53.3	49.7–56.7	366	46.7	43.3–50.3	< 0.001
High	237	42.6	38.6–46.8	319	57.4	53.2–61.4	
Gini Index							
High	165	54.5	48.8–60.0	138	45.5	40.0–51.2	0.026
Low	489	47.2	44.2–50.3	547	52.8	49.8–55.8	

*Pearson’s Chi-square test.

## DISCUSSION

This study showed an association between the potential support of the school environment for oral health promotion, as measured by the AEPSB indicator, and contextual factors related to schools, capital cities and geographic regions. Hitherto, the scientific literature presents no evidence of another study that has proposed an indicator of this nature, with the aim of specifically assessing aspects related to oral health in schools.

Socioeconomic inequalities related to human development were associated with school environments with potential for oral health promotion. Private schools located in capitals with lower HDI or higher Gini index had lower prevalence of oral health promoting environments . Studies in Brazil^8^ , Ireland^11^ and Canada^16 , 17^ have shown the influence of contextual socioeconomic aspects – referring to the municipality or the school environment – in the health of students.

Most schools with a higher AEPSB are concentrated, therefore, on the public sector and in capitals and regions with better socioeconomic indicators, especially in the South and Southeast. Regarding the capitals and regions, these results were already expected, considering that these indicators presuppose better organization of school services, with greater attention to the aspects of health promotion at school. Similarly, in the study by Horta et al.^22^ , which classified the schools of PeNSE according to the potential for general health promotion, the schools in the South and Southeast regions also obtained the highest overall scores, whereas the Northeast region obtained the lowest score. Another study conducted in Brazil, which evaluated the effectiveness of strategies to promote oral health in the context of the primary health care, showed similar interregional inequalities, with better performances in the South and Southeast regions, in contrast with the North, Northeast and Midwest regions^30^ . These findings reinforce the need for greater institutional attention, based on the health promotion principles, in search of strategic actions aimed at these regions. The social determinants of health are of vital importance to effectively reduce evident inequalities – not only in schools, but also in health services.

With reference to the highest percentage of higher AEPSB in public schools, we consider that the implementation of public policies related to adolescent’s school health – for example, the *Programa Saúde na Escola* (Health in School Program)^31^ , the *Programa Nacional de Alimentação Escolar* (National Program of School Diet)^32^ and the *Política Nacional de Promoção da Saúde* (National Policy of Health Promotion)^33^ – happens unevenly between public and private schools, being more effective in the former, due to the students’ greater health need and also greater governmental control over these institutions. Horta et al.^22^ , however, verified a considerably higher score in private schools in relation to the general health promoting environment. It is noteworthy, however, that the indicator designed in their study (the EPSAE)^22^ involved no specific variables of oral health, given the difference in scope. These differences may explain part of the discordances observed in this specific result. In any case, there is a need for further studies on the factors regarding the schools’ potential for oral and general health promotion.

The use of data from a national survey with a representative sample of schools is one of the strengths of our study. Moreover, using a methodology still little used in this field – CATPCA – it was possible to develop the indicator AEPSB, which can be used in other analyses to know its influence on students’ oral health.

A frequent recommendation for the implementation of healthy school environments refers to the availability of foods with lower cariogenic potential, as well as the restriction of foods with greater potential^17 , 18 , 34 , 35^ . The regular availability of beverages with added sugar in the school, such as soft drinks, has been associated with a higher daily consumption of these drinks by adolescents^35^ and a high prevalence of caries^18^ . The AEPSB indicator covered these aspects. Of the ten variables that comprised the final model, six referred to the sale of foods with added sugar, not only in the school canteen, but also in alternative points in the school neighborhood.

As in the study by Horta et al.^22^ , one of the limitations of this analysis refers to the reduced number of variables for the composition of the indicator. The characterization of a school environment as a possible oral health promoting environment certainly goes beyond the explored spectrum. For a better evaluation, it becomes relevant to include other aspects not addressed in this national survey: the involvement of the school with the community and the inclusion of oral health promotion in the school curriculum , in addition to specific structural and processual aspects that would favor oral health – for example, the presence of places suitable for oral hygiene (known as *escovódromos* ), the existence of environments with lower risk for dental trauma and the performance of frequent and scheduled activities for oral health education^8 , 9^ . We would suggest that future editions of PeNSE incorporate questions related to these dimensions.

The use of contextual data related to the capital cities and regions (HDI and Gini index) with a five-year gap may have implications for the results of the study, because they reflect situations at different points in time. Data from 2015 would be more appropriate, but were not available. This limitation is inherent to studies that use secondary data.

Another limitation refers to the percentage of explanation resulting from our CATPCA analysis. We obtained a considerably acceptable value (above half), but it would be desirable that it was closer to the maximum value. Similarly, the total Cronbach’s alpha coefficient of the aforementioned analysis brings a very permissible result, the set of the three dimensions generated are considered^25^ . However, this did not occur when the dimensions were analyzed separately, and coefficients below 0.7 were found. Despite these limitations, the results indicate a possible validity of the proposed indicator, considering the coherence and plausibility verified. We emphasize that the CATPCA analysis is exploratory and presupposes other studies with the objective of testing the instrument validity.

We conclude that the AEPSB indicator was associated with contextual factors of the regions, capital cities and schools, being higher in regions and capitals with greater human development (HDI) and lower socioeconomic inequality (Gini) and in public schools. Additional studies should be performed to verify these associations in other contexts.

Our findings suggest the need to broaden public policies in Brazil, seeking to improve school environments in terms of political, curricular, pedagogical, structural and relational aspects that can contribute, directly or indirectly, for oral health promotion. Strategic institutional actions that consider regional socioeconomic diversities are relevant and necessary to reduce existing inequalities.

The AEPSB indicator developed for this analysis using categorical variables related to the school environment may constitute an appropriate tool to assess the potential of oral health promotion in schools. It may be adapted to other contexts but requires further validation studies. The data analysis technique employed (CATPCA) for the development of the aforementioned indicator can be replicated in other studies with similar objectives, and may include additional variables to broaden and favor the assessments.
