# Supplementary material for: School environment and oral health promotion: the National Survey of School Health (PeNSE)
Source: Rev Saude Publica. 2019 Oct 16;53:93. doi: 10.11606/s1518-8787.2019053001376 (PMC6802948; doi:10.11606/s1518-8787.2019053001376)
Supplement: Translation

# Translation: Ambiente escolar e promoção de saúde bucal: a Pesquisa Nacional de Saúde do Escolar (PeNSE)

**Authors:** Newillames Gonçalves Nery, Lidia Moraes Ribeiro Jordão, Maria do Carmo Matias Freire

**Affiliations:** I Secretaria Municipal de Saúde de Goiânia. Goiânia, GO, Brasil; II Universidade Federal de Goiás. Faculdade de Odontologia. Programa de Pós-Graduação em Odontologia. Goiânia, GO, Brasil

**Keywords:** Saúde do Adolescente, Serviços de Odontologia Escolar, Serviços de Saúde Escolar, Disparidades nos Níveis de Saúde, Saúde Bucal, Promoção da Saúde, Inquéritos Epidemiológicos

## Abstract

**OBJETIVO:**

Avaliar o potencial de suporte do ambiente escolar para a promoção da saúde bucal e fatores associados nas capitais brasileiras.

**MÉTODOS:**

Os dados de 1.339 escolas públicas e privadas das 27 capitais brasileiras foram obtidos da Pesquisa Nacional de Saúde do Escolar (PeNSE) 2015. Os dados das capitais foram obtidos do Programa das Nações Unidas para o Desenvolvimento e do Datasus. Foi elaborado o indicador “ambiente escolar promotor de saúde bucal” (AEPSB), a partir de 21 variáveis do ambiente escolar com possível influência na saúde bucal dos escolares empregando a análise de componentes principais para dados categóricos (CATPCA). Associações entre o AEPSB e características das escolas, das capitais e das regiões foram testadas (análises bivariadas).

**RESULTADOS:**

Dez variáveis compuseram a CAPTCA, após exclusão daquelas com baixa correlação ou alta multicolinearidade. A análise resultou em modelo com três dimensões: D1. aspectos intraescolares (venda de alimentos com açúcar adicionado na cantina e ações de promoção de saúde), D2. aspectos do entorno escolar (venda de alimentos com açúcar adicionado em pontos alternativos) e D3. políticas proibitivas na escola (proibição do consumo de álcool e tabaco). A soma dos escores das dimensões gerou o indicador AEPSB, dicotomizado pela mediana. Do total de escolas estudadas, 51,2% (IC95% 48,5–53,8) apresentaram ambiente mais favorável à saúde bucal (maior AEPSB). Nas capitais, esse percentual variou de 36,6% (IC95% 23,4–52,2) no Rio Branco a 80,4% (IC95% 67,2–89,1) em Florianópolis. Entre as regiões brasileiras, variou de 45,5% (IC95% 40,0–51,2), no Norte a 67,6% (IC95% 59,4–74,9) no Sul. Percentuais maiores de escolas com maior AEPSB foram encontrados na rede pública [58,1% (IC95% 54,9–61,2)] e em capitais e regiões com maior índice de desenvolvimento humano [61,0% (IC95% 55,8–66,0) e 57,4% (IC95% 53,2–61,4), respectivamente] e menor índice de Gini [55,7% (IC95% 51,2–60,0) e 52,8 (IC95% 49,8–55,8), respectivamente].

**CONCLUSÕES:**

O potencial de suporte à promoção da saúde bucal de escolas das capitais brasileiras, avaliado pelo indicador AEPSB, foi associado a fatores contextuais das escolas, das capitais e das regiões brasileiras.

## INTRODUÇÃO

Os ambientes em que as pessoas vivem são componentes importantes da promoção da saúde^1^ . Fatores ambientais, além dos individuais, podem influenciar a relação entre educação e saúde^2^ . A escola é um dos espaços estratégicos para o estímulo e desenvolvimento de habilidades, comportamentos e estilos de vida mais saudáveis, particularmente entre crianças e adolescentes^2 , 3^ . Nesse sentido, estudos têm investigado fatores ambientais – físicos ou sociais – e intervenções promotoras de saúde realizadas na escola, e sua associação com condições, percepções e comportamentos relacionados à saúde dos escolares^4^ .

Na década de 1990, a Organização Mundial da Saúde, reconhecendo esse aspecto na relação intrínseca entre educação e saúde, sugeriu a abordagem denominada “escolas promotoras de saúde” (EPS), baseada em ambientes escolares ditos “saudáveis”^7^ . Nesses ambientes, políticas institucionais de saúde, currículo escolar, ambiente social e físico e a interação com a comunidade, somados a práticas indutoras de autoestima e de saúde dos indivíduos, podem proporcionar apoio ou suporte à promoção da saúde^7^ .

Estudos de revisão sobre a efetividade da abordagem das EPS, assim como de outros modelos para a promoção da saúde na escola, revelam que os resultados das ações são variáveis e limitados, mas com potenciais efeitos positivos na saúde individual e coletiva, assim como no desempenho acadêmico^4^ . Programas de promoção da saúde no ambiente escolar com maior tempo de duração e maior envolvimento da comunidade escolar, bem como aqueles que abordam saúde mental, alimentação saudável e atividades físicas, apresentam maiores evidências de efeitos positivos^12^ . Adicionalmente, a disponibilidade de opções alimentares mais saudáveis dentro e no entorno das escolas, bem como a restrição ao uso do tabaco e álcool, interferem positivamente no estabelecimento de hábitos preventivos de doenças crônicas não transmissíveis^13^ .

A relação entre ambiente escolar e saúde bucal também tem sido investigada, porém com menor quantidade de estudos^14^ . No Brasil, ambientes escolares favoráveis à promoção da saúde foram associados a melhores condições de saúde bucal, com menores prevalências de cárie e traumatismo dentário^8 , 9 , 14^ , além de melhor qualidade de vida relacionada à saúde bucal^15^ . Essas associações têm sido estudadas também em outros países. No Canadá, foi observada associação entre ambiente socioeconômico escolar mais desfavorável e maior prevalência de dor e cárie dentária em escolas públicas^16^ , além da associação entre ambientes escolares com potencial de promoção da saúde bucal e menor incidência de cárie^17^ . Na Tailândia, a disponibilidade de alimentos saudáveis nos espaços escolares foi associada ao menor consumo de doces e índices de cárie mais baixos^18^ . Uma revisão sistemática recente, envolvendo estudos em âmbito mundial que avaliaram as ações de promoção da saúde e prevenção de fatores de risco no contexto das EPS, demonstrou a inexistência de atividades específicas no ambiente escolar voltadas para a promoção da saúde bucal^19^ .

A necessidade de instrumentos consolidados para avaliar as ações relacionadas à promoção da saúde bucal, não somente no ambiente escolar, mas também em outros cenários, constitui um importante desafio metodológico e estratégico a ser superado^20^ . A construção de indicadores utilizando dados de inquéritos nacionais de saúde do escolar pode constituir uma alternativa apropriada para contribuir para esses processos avaliativos. A Pesquisa Nacional de Saúde do Escolar (PeNSE), realizada trienalmente desde 2009 pelo Ministério da Saúde e pelo Instituto Brasileiro de Geografia e Estatística (IBGE), busca contribuir para o acompanhamento da situação de saúde dos adolescentes escolares brasileiros. Esse inquérito abrange diversas questões relativas à saúde e comportamentos individuais, além de variáveis referentes ao ambiente da escola, as quais podem exercer influência sobre a saúde geral e bucal dos estudantes^21^ .

A partir dos dados da PeNSE de 2015, Horta et al.^22^ elaboraram o escore de promoção de saúde no ambiente escolar (EPSAE), o qual identificou ambientes escolares com melhores condições de promoção da saúde. Embora muitos fatores de risco para as doenças e agravos bucais sejam comuns para outras doenças crônicas^23^ , é relevante considerar as características das escolas que estão mais diretamente relacionadas à promoção da saúde bucal, possibilitando o planejamento e a avaliação de ações específicas.

Assim, este estudo objetivou avaliar o potencial de suporte à promoção da saúde bucal no ambiente escolar das capitais brasileiras e fatores associados.

## MÉTODOS

Estudo transversal utilizando o banco de dados da PeNSE 2015, disponível no *site* do IBGE^24^ . A PeNSE 2015 utilizou plano amostral complexo (por conglomerados), envolvendo aproximadamente 102.072 estudantes adolescentes do nono ano do ensino fundamental de 3.040 escolas públicas e privadas das 27 capitais brasileiras e do interior. As escolas foram selecionadas a partir de informações do Censo Escolar de 2013, que constituía o cadastro mais atualizado no momento do planejamento da pesquisa. Foi garantida a presença de escolas públicas (federais, estaduais e municipais) e privadas na amostra, em proporção aproximadamente semelhante à existente no cadastro de seleção.

Para o presente estudo, foram utilizados apenas os dados referentes às 1.339 escolas públicas e privadas das 26 capitais brasileiras e do Distrito Federal. O instrumento de coleta desses dados foi um questionário eletrônico estruturado, aplicado por meio de entrevistas com os diretores ou responsáveis pelas instituições. O projeto da PeNSE 2015 foi aprovado pelo Conselho Nacional de Ética em Pesquisa (parecer 1.006.467/2015). Mais informações sobre os aspectos metodológicos desse inquérito podem ser obtidas em publicação anterior^21^ .

Inicialmente, foi elaborado o indicador “ambiente escolar promotor de saúde bucal” (AEPSB), destinado a caracterizar o potencial de suporte do ambiente escolar para a promoção de saúde bucal. Na primeira etapa, três profissionais com conhecimento e experiência em saúde bucal coletiva selecionaram, por consenso, 21 questões do questionário da PeNSE 2015 com potencial de influenciar condições ou comportamentos relativos à saúde bucal dos adolescentes^21^ . As variáveis selecionadas foram: “venda de refrigerantes na cantina”, “venda de outras bebidas com açúcar adicionado na cantina”, “venda de balas e outras guloseimas na cantina”, “venda de frutas frescas ou saladas de frutas na cantina”, “venda de refrigerantes em pontos alternativos”, “venda de outras bebidas com açúcar adicionado em pontos alternativos”, “venda de balas e outras guloseimas em pontos alternativos”, “venda de frutas frescas ou saladas de frutas em pontos alternativos”, “escola possui horta”, “escola possui pia em condições de uso”, “escola possui grupo ou comitê de saúde”, “escola aderiu ao Programa Saúde na Escola (PSE)”, “escola desenvolve ações do PSE”, “escola desenvolve ações do Programa Mais Educação”, “escola realiza ações em conjunto com a unidade básica de saúde (UBS)”, “escola possui registro sobre informações de saúde dos alunos”, “escola possui materiais e/ou medicamentos de primeiros socorros”, “escola tem conhecimento de professores que fumam na escola”, “escola tem conhecimento de alunos que fumam na escola”, “escola proíbe consumo do tabaco” e “escola proíbe consumo de bebidas alcoólicas”.

Todas essas variáveis estavam categorizadas inicialmente em sim ou não, exceto “Escola possui pia em condições de uso” (sim, não, não está em condições de uso) e “Escola possui materiais ou medicamentos de primeiros socorros” (sim, não, não está em local adequado). Para este estudo, ambas foram categorizadas como sim ou não da seguinte forma: “não está em condições de uso” foi considerada não para a primeira variável, e “não está em local adequado” foi considerado sim para a segunda.

Após análise das variáveis selecionadas em matriz de correlação, 11 foram excluídas, eliminando aquelas com baixa correlação (todos os valores de correlação < 0,4) ou alta multicolinearidade (algum valor de correlação > 0,9). Assim, dez variáveis compuseram a análise final: “venda de refrigerantes na cantina”, “venda de outras bebidas com açúcar adicionado na cantina”, “venda de balas e outras guloseimas na cantina”, “venda de refrigerantes em pontos alternativos”, “venda de outras bebidas com açúcar adicionado em pontos alternativos”, “venda de balas e outras guloseimas em pontos alternativos”, “escola desenvolve ações do PSE”, “escola realiza ações em conjunto com a UBS”, “escola proíbe consumo do tabaco” e “escola proíbe consumo de bebidas alcoólicas”.

Em seguida, foi realizada a redução dessas dez variáveis, buscando construir um indicador que as resumisse. A análise de componentes principais para dados categóricos (CATPCA) ou não lineares foi utilizada. A partir dela, obtêm-se resultados comparáveis à análise de componentes principais (PCA), a qual é geralmente utilizada para variáveis numéricas^25^ . Esse método é utilizado para redução de variáveis categóricas (nominais ou ordinais), destinando-se a diminuir a dimensionalidade dos dados, resumindo um grande número de variáveis em alguns componentes (dimensões) não correlacionados, com a menor perda de informação possível. As categorias das variáveis recebem valores numéricos por meio de um processo denominado quantificação, escala ou pontuação ótima. Com esses valores numéricos, a variância das variáveis é contabilizada^25^ .

Assim, valores próprios são gerados, indicando: a variação contabilizada por componente ou dimensão; a carga dos componentes (que reflete as correlações entre variáveis quantificadas e componentes principais); as somas das cargas (autovalores ou *eingenvalues* ), as quais revelam as contribuições das variáveis para a variância total dos dados (percentual de explicação) e os escores para cada componente, que podem ser úteis em outras etapas analíticas. Na síntese dos dados, é apresentado o coeficiente alfa de Cronbach, o qual quantifica, em uma escala de zero a um, a confiabilidade e consistência interna dos dados obtidos em questionário ou escala, sendo comumente desejável um valor acima de 0,7^25^ . Nesta análise, os valores não informados, perdidos ou faltantes ( *missing* ) foram imputados com a moda da variável quantificada. Utilizou-se o *software* estatístico SPSS (versão 23, SPSS Inc., Chicago, IL, USA). Ao final da CATPCA, os escores foram somados e, devido à inexistência de um valor de referência e da distribuição não normal dos dados, a variável resultante foi dicotomizada com base na mediana, gerando o indicador AEPSB.

Na etapa posterior, foram realizadas análises descritivas e bivariadas (testes do qui-quadrado), buscando identificar as características das escolas, capitais e regiões associadas ao indicador de promoção de saúde bucal (AEPSB). As variáveis das escolas foram suas características geográficas e organizacionais: localização da escola no município (rural ou urbana), dependência administrativa (pública ou privada), e regime escolar integral (não ou sim). As variáveis quantitativas relativas às capitais foram categorizadas com base nos tercis de índice de desenvolvimento humano (IDH) (baixo, médio ou alto) e índice de Gini (alto, médio ou baixo). Para as regiões, os parâmetros foram: IDH (baixo ou alto) e índice de Gini (alto ou baixo). O nível de significância estatística foi estabelecido em 5%.

O IDH é calculado a partir de indicadores de educação, de longevidade e de renda da população, variando de zero a um. Quanto mais próximo de um, maior o desenvolvimento humano populacional do município e da região^26^ . O índice de Gini afere a desigualdade na distribuição de renda e também varia de zero a um, porém com interpretação diferente: quanto mais próximo de um, maior a desigualdade econômica na população^27^ . Os dados relativos ao IDH foram obtidos no banco de dados do Atlas do Desenvolvimento Humano no Brasil^28^ , enquanto os valores de índice de Gini foram coletados na base de dados institucional do Datasus^29^ . Ambos são referentes ao ano de 2010, que é o ano com dados disponíveis mais próximo da PeNSE 2015.

## RESULTADOS

A Tabela 1 apresenta a distribuição das escolas segundo situação geográfica por regiões, capitais e características organizacionais. A maioria das escolas estudadas eram localizadas em zona urbana, pública e com regime não integral. A matriz de correlação final, incluindo as dez variáveis que foram analisadas a partir da CATPCA para a geração do indicador AEPSB, encontra-se na Tabela 2 .

**Tabela 1:** Distribuição das escolas segundo localização geográfica por regiões e capitais e características organizacionais, com base na Pesquisa Nacional de Saúde do Escolar (PeNSE) 2015.

Localização geográfica	n	%
Regiões e capitais		
Norte	303	22,6
Porto Velho (RO)	46	3,4
Rio Branco (AC)	41	3,1
Manaus (AM)	35	2,6
Boa Vista (RR)	44	3,3
Belém (PA)	46	3,4
Macapá (AP)	51	3,8
Palmas (TO)	40	3,0
Nordeste	480	35,9
São Luís (MA)	54	4,0
Teresina (PI)	58	4,3
Fortaleza (CE)	46	3,4
Natal (RN)	59	4,4
João Pessoa (PB)	68	5,1
Recife (PE)	56	4,2
Maceió (AL)	41	3,1
Aracaju (SE)	54	4,0
Salvador (BA)	44	3,3
Sudeste	218	16,3
Belo Horizonte (MG)	62	4,6
Vitória (ES)	62	4,6
Rio de Janeiro (RJ)	51	3,8
São Paulo (SP)	43	3,2
Sul	139	10,4
Curitiba (PR)	42	3,1
Florianópolis (SC)	51	3,8
Porto Alegre (RS)	46	3,4
Centro-Oeste	199	14,9
Campo Grande (MS)	44	3,3
Cuiabá (MT)	39	2,9
Goiânia (GO)	68	5,1
Distrito Federal (DF)	48	3,6

Total	1.339	100,0

Características organizacionais		

Localização no município		
Rural	48	3,6
Urbana	1.291	96,4
Total	1.339	100,0
Dependência administrativa		
Pública	940	70,2
Privada	399	29,8
Total	1.339	100,0
Regime escolar integral		
Sim	275	20,5
Não	1.063	79,4
Total	1.338	100,0

**Tabela 2:** Matriz de correlações final com as dez variáveis relacionadas ao ambiente escolar promotor de saúde bucal, selecionadas para a análise de componentes principais para dados categóricos (CATPCA), com base na Pesquisa Nacional de Saúde do Escolar (PeNSE) 2015.

Variáveis		Venda de refrigerantes na cantina	Venda de outras bebidas com açúcar adicionado na cantina	Venda de balas e outras guloseimas na cantina	Venda de refrigerantes em pontos alternativos	Venda de outras bebidas com açúcar adicionado em pontos alternativos	Venda de balas e outras guloseimas em pontos alternativos	Escola desenvolve ações do PSE	Escola realiza ações em conjunto com a UBS	Escola proíbe consumo do tabaco	Escola proíbe consumo de bebidas alcoólicas
Venda de refrigerantes na cantina	Correlação de Pearson	1									
	p										
	n	1.304									
Venda de outras bebidas com açúcar adicionado na cantina	Correlação de Pearson	**0,50**	1								
	p	**< 0,001**									
	n	1.304	1.304								
Venda de balas e outras guloseimas na cantina	Correlação de Pearson	**0,60**	**0,47**	1							
	p	**< 0,001**	**< 0,001**								
	n	1.304	1.304	1.304							
Venda de refrigerantes em pontos alternativos	Correlação de Pearson	-0,02	0,02	-0,01	1						
	p	0,433	0,509	0,778							
	n	1.300	1.300	1.300	1.332						
Venda de outras bebidas com açúcar adicionado em pontos alternativos	Correlação de Pearson	-0,00	**0,08**	**0,06**	**0,63**	1					
	p	0,857	**0,005**	**0,023**	**< 0,001**						
	n	1.300	1.300	1.300	1.332	1.332					
Venda de balas e outras guloseimas em pontos alternativos	Correlação de Pearson	0,00	0,04	**0,11**	**0,53**	**0,46**	1				
	p	0,926	0,121	**< 0,001**	**< 0,001**	**< 0,001**					
	n	1.300	1.300	1.300	1.332	1.332	1.332				
Escola desenvolve ações do PSE	Correlação de Pearson	**0,27**	**0,23**	**0,22**	0,03	**0,07**	0,03	1			
	p	**< 0,001**	**< 0,001**	**< 0,001**	0,298	**0,013**	0,271				
	n	1.303	1.303	1.303	1.331	1.331	1.331	1.336			
Escola realiza ações em conjunto com a UBS	Correlação de Pearson	**0,25**	**0,22**	**0,18**	0,004	0,035	-0,026	**0,40**	1		
	p	**< 0,001**	**< 0,001**	**< 0,001**	0,873	0,200	0,350	**< 0,001**			
	n	1.303	1.303	1.303	1.331	1.331	1.331	1.335	1.336		
Escola proíbe consumo do tabaco	Correlação de Pearson	0,02	-0,01	0,01	-0,01	**-0,05**	-0,03	**0,05**	**0,07**	1	
	p	0,563	0,617	0,632	0,623	**0,050**	0,303	**0,047**	**0,013**		
	n	1.303	1.303	1.303	1.331	1.331	1.331	1.335	1.335	1.336	
Escola proíbe consumo de bebidas alcoólicas	Correlação de Pearson	0,01	-0,04	-0,01	-0,04	**-0,06**	**-0,07**	0,032	0,032	**0,60**	1
	p	0,684	0,149	0,755	0,141	**0,023**	**0,017**	0,240	0,238	**0,000**	
	n	1.303	1.303	1.303	1.331	1.331	1.331	1.335	1.335	1.336	1.336

p: Significância bilateral.

PSE: Programa Saúde na Escola; UBS: unidade básica de saúde.

Os resultados referentes à elaboração do indicador AEPSB encontram-se na Tabela 3 . A CATPCA resultou em um modelo com três dimensões, com percentual de explicação da variância dos dados aceitável (61,2%) e alto coeficiente alfa de Cronbach (0,93).

**Tabela 3:** Resultados da análise de componentes principais para dados categóricos (CATPCA) para as variáveis relacionadas ao ambiente escolar promotor de saúde bucal, com base na Pesquisa Nacional de Saúde do Escolar (PeNSE) 2015.

Variáveis e dimensões resultantes	n	%	Análise CATPCA				

			Variância contabilizada	Cargas fatoriais	Autovalor	Percentual de explicação da variância	Alfa de Cronbach
Dimensão 1 – Aspectos intraescolares							

Cantina: refrigerantes			0,566	0,752	2,419	24,2%	0,652
Sim	404	30,2					
**Não**	900	67,2					
Cantina: balas ou guloseimas			0,546	0,739			
Sim	324	24,2					
**Não**	980	73,2					
Cantina: bebidas com açúcar adicionado			0,499	0,706			
Sim	284	30,2					
**Não**	1.020	76,2					
Escola: ações do PSE			0,302	0,549			
Não	796	59,4					
**Sim**	540	40,3					
Escola: ações com a UBS			0,250	0,500			
Não	840	62,7					
**Sim**	496	37,0					

Dimensão 2 – Aspectos do entorno escolar							

Ponto alternativo: refrigerantes			0,656	0,810	2,099	21,0%	0,582
Sim	276	20,6					
**Não**	1.056	78,9					
Ponto alternativo: outras bebidas com açúcar adicionado			0,595	0,771			
Sim	162	12,1					
**Não**	1.170	87,4					
Ponto alternativo: balas ou guloseimas			0,517	0,719			
Sim	244	18,2					
**Não**	1.088	81,3					

Dimensão 3 – Políticas proibitivas							

Escola: proíbe consumo de tabaco			0,744	0,863	1,602	16,0%	0,417
Não	1.214	90,7					
**Sim**	111	8,3					
Escola: proíbe consumo de álcool			0,720	0,849			
Não	1.225	91,5					
**Sim**	111	8,3					

Total			-	-	6,120	61,2%	0,930

PSE: Programa Saúde na Escola; UBS: unidade básica de saúde.

Notas: São destacadas em negrito as características ambientais potencialmente favoráveis à saúde bucal.

Os valores relativos à categoria “não informado” de cada variável foram considerados nas análises, mas omitidos na tabela, pelo baixo percentual relativo (entre 0,2 e 2,6%).

O percentual de explicação da variância é calculado a partir do quociente entre o autovalor ( *eingenvalue* ) e o número total de variáveis (neste caso, dez).

As três dimensões geradas foram: dimensão 1. aspectos intraescolares – venda de alimentos com adição de açúcar na cantina da escola (refrigerantes, outras bebidas com açúcar adicionado, balas e outras guloseimas) e ações de promoção de saúde realizadas na escola (ações do PSE ou ações em conjunto com a UBS); dimensão 2. aspectos do entorno escolar – venda de alimentos com adição de açúcar em pontos alternativos localizados próximos à escola (refrigerantes, outras bebidas com açúcar adicionado, balas e outras guloseimas); e dimensão 3. políticas proibitivas – proibição interna do consumo de álcool e tabaco pela escola ( Tabela 3 ).

Para cada dimensão, a CATPCA gerou um escore para o ambiente escolar. Para a dimensão 1, o escore variava de -2,85 a 1,43, com mediana 0,25; para a dimensão 2, variava de -3,86 a 1,96, com mediana 0,22; para a dimensão 3, variava de -4,57 a 1,12, com mediana 0,34. Os escores das três dimensões foram somados, resultando em um escore total que variou de -7,46 a 2,07, tendo como mediana o valor 0,64. Esse escore total foi dicotomizado pela mediana, gerando o indicador geral do potencial de promoção de saúde bucal, cujas categorias foram: maior potencial (escola com escore geral acima da mediana, ou seja, maior AEPSB – de 0,64 a 2,07) e menor potencial (escola com escore geral abaixo da mediana, ou seja, menor AEPSB – de -7,46 a 0,64).

Utilizando o AEPSB, 685 escolas (51,2%; IC95% 48,5–53,8) apresentaram ambiente escolar com maior potencial de promoção de saúde bucal. As frequências em cada uma das 27 capitais e das cinco regiões brasileiras encontram-se na Figura 1 , apresentando grandes variações. As regiões Norte e Nordeste apresentaram menores proporções de ambientes escolares com maior AEPSB, enquanto a região Sul se destacou com os maiores percentuais (p < 0,001). Dentre as capitais, Florianópolis (80,39%; IC95% 67,24–89,12) e Belo Horizonte (67,7%; IC95% 55,2–78,2) apresentaram maiores percentuais de escolas com maior AEPSB, enquanto Belém (34,8%; IC95% 22,5–49,5) e Recife (35,7%; IC95% 24,3–49,0) apresentaram os menores percentuais (p < 0,001).

**Figura 1:** Distribuição dos percentuais das escolas, segundo o indicador “ambiente escolar promotor de saúde bucal” (AEPSB), por capitais e regiões, com base na Pesquisa Nacional de Saúde do Escolar (PeNSE) 2015.

Nota: Teste qui-quadrado de Pearson (p < 0,001)

Houve associações significantes entre o potencial de promoção da saúde bucal e as variáveis relacionadas aos contextos das capitais e das regiões ( Tabela 4 ): escolas com maior AEPSB foram mais frequentes nas capitais e regiões com maior IDH (p < 0,001) e menor índice de Gini (p < 0,05). Escolas com maior AEPSB foram mais frequentes na rede pública (58,1%; IC95% 55,0–61,2) do que na rede privada (34,8%; IC95% 30,3–39,7) (p < 0,001).

**Tabela 4:** Distribuição das escolas segundo o indicador “ambiente escolar promotor de saúde bucal” (AEPSB) e características das escolas e capitais, com base na Pesquisa Nacional de Saúde do Escolar (PeNSE) 2015.

Nível	Escolas com menor AEPSB (n = 654)			Escolas com maior AEPSB (n = 685)			p*

Variáveis							
		
Categorias	n	%	IC95%	n	%	IC95%	
Escola							
Localização da escola							
Rural	22	45,8	32,4–59,9	26	54,2	40,1–67,6	0,671
Urbana	632	48,9	46,2–51,7	659	51,1	48,3–53,8	
Dependência administrativa							
Pública	394	41,9	38,8–45,1	546	58,1	54,9–61,2	< 0,001
Privada	260	65,2	60,4–69,7	139	34,8	30,3–39,7	
Regime integral							
Não	525	49,4	46,4–52,4	538	50,6	47,6–53,6	0,401
Sim	128	46,5	40,7–52,5	147	53,5	47,5–59,3	
Capital							
Índice de desenvolvimento humano							
Baixo	246	54,4	49,8–59,0	206	45,6	41,0–50,2	
Médio	270	50,7	46,4–54,9	263	49,3	45,1–53,6	< 0,001
Alto	138	39,0	34,0–44,1	216	61,0	55,8–66,0	
Índice de Gini							
Alto	201	54,0	48,9–59,0	171	46,0	41,0–51,0	
Médio	237	49,4	44,9–53,9	243	50,6	46,2–55,1	0,018
Baixo	216	44,4	40,0–48,8	271	55,7	51,2–60,0	
Região							
Índice de desenvolvimento humano							
Baixo	417	53,3	49,7–56,7	366	46,7	43,3–50,3	< 0,001
Alto	237	42,6	38,6–46,8	319	57,4	53,2–61,4	
Índice de Gini							
Alto	165	54,5	48,8–60,0	138	45,5	40,0–51,2	0,026
Baixo	489	47,2	44,2–50,3	547	52,8	49,8–55,8	

*Teste qui-quadrado de Pearson

## DISCUSSÃO

O presente estudo mostrou que há associação entre o potencial de suporte do ambiente escolar para a promoção da saúde bucal, analisado pelo indicador AEPSB, e fatores contextuais relativos às escolas, às capitais e às regiões geográficas. Na literatura científica publicada até o momento, não há evidência de outro estudo que tenha proposto um indicador desta natureza, com a finalidade de avaliar especificamente aspectos relacionados à saúde bucal em escolas.

Desigualdades socioeconômicas e relativas ao desenvolvimento humano foram associadas aos ambientes escolares com potencial de promoção da saúde bucal. Escolas privadas e localizadas em capitais com menor IDH ou maior índice de Gini apresentaram menores prevalências de ambientes escolares promotores de saúde bucal. Estudos no Brasil^8^ , na Irlanda^11^ e no Canadá^16 , 17^ têm mostrado a influência de aspectos contextuais socioeconômicos – referentes ao município ou ao ambiente da escola – na saúde dos escolares.

A maior parte das escolas com maior AEPSB se concentra, portanto, na rede pública e em capitais e regiões com melhores indicadores socioeconômicos, especialmente no Sul e Sudeste. Com relação às capitais e regiões, esses resultados já eram esperados, considerando-se que esses indicadores pressupõem melhor organização dos serviços escolares, com maior atenção aos aspectos de promoção da saúde na escola. De forma semelhante, no estudo de Horta et al.^22^ , que classificou as escolas da PeNSE de acordo com o potencial de promoção de saúde geral, as escolas das regiões Sul e Sudeste também obtiveram os maiores escores totais, enquanto a região Nordeste obteve a menor pontuação. Outro estudo realizado no Brasil, que avaliou a efetividade de estratégias de promoção da saúde bucal no âmbito da atenção primária em saúde, revelou desigualdades inter-regionais semelhantes, com melhores desempenhos nas regiões Sul e Sudeste, em contraste com as regiões Norte, Nordeste e Centro-Oeste^30^ . Esses achados reforçam a necessidade de maior atenção institucional, pautada nos princípios da promoção da saúde, em busca de ações estratégicas voltadas a essas regiões. É, também, de vital importância considerar os determinantes sociais da saúde, para efetiva redução das iniquidades evidentes – não somente nas escolas, mas também nos serviços de saúde.

Com referência ao percentual mais elevado de maior AEPSB nas escolas públicas, entendemos que a implantação de políticas públicas relacionadas à saúde do escolar adolescente – por exemplo, o Programa Saúde na Escola^31^ , o Programa Nacional de Alimentação Escolar^32^ e a Política Nacional de Promoção da Saúde^33^ – acontece de forma desigual entre escolas públicas e privadas, sendo mais efetivas nas primeiras, devido tanto à maior necessidade dos estudantes quanto à maior atenção e controle governamental sobre essas instituições. Horta et al.^22^ , no entanto, verificaram uma pontuação consideravelmente maior nas escolas privadas em relação ao ambiente promotor de saúde geral. Ressalta-se, entretanto, que o indicador construído em seu estudo (o EPSAE)^22^ não envolveu variáveis específicas da saúde bucal, dada a diferença no escopo. Essas diferenças podem explicar parte das discordâncias observadas neste resultado específico. De toda forma, percebe-se a necessidade de estudos mais aprofundados acerca dos fatores que caracterizam o potencial promotor de saúde bucal e geral nesses cenários.

A utilização de dados de um inquérito nacional com amostra representativa de escolas é um dos pontos fortes do presente estudo. Ademais, por meio de uma metodologia ainda pouco utilizada neste campo, a CATPCA, foi possível construir o indicador AEPSB, que poderá ser utilizado em outras análises, com o objetivo de conhecer a sua influência na saúde bucal dos escolares.

Uma recomendação frequente para a implementação de ambientes escolares saudáveis refere-se à disponibilização de alimentos com menor potencial cariogênico, assim como à restrição de alimentos com maior potencial^17 , 18 , 34 , 35^ . A disponibilidade regular de bebidas com adição de açúcar na escola, como os refrigerantes, tem sido associada ao maior consumo diário deles por adolescentes^35^ e à alta prevalência de cárie^18^ . O indicador AEPSB contemplou esses aspectos. Das dez variáveis que compuseram o modelo final, seis se referiam à venda de alimentos com adição de açúcar, não somente na cantina da escola, como também em pontos alternativos na vizinhança escolar.

Assim como no estudo de Horta et al.^22^ , uma das limitações desta análise se refere ao reduzido número de variáveis para a composição do indicador. A caracterização de um ambiente escolar como possível promotor de saúde bucal, certamente, vai além do espectro explorado. Para uma melhor avaliação, torna-se relevante incluir outros aspectos não abordados nesse inquérito nacional: o envolvimento da escola com a comunidade e o currículo escolar contemplando a temática da promoção da saúde bucal, além de aspectos estruturais e processuais específicos que favoreçam a saúde bucal – por exemplo, a presença de locais apropriados para a higiene bucal (conhecidos como escovódromos), a existência de ambientes com menor risco para traumatismos bucais e a realização de atividades frequentes e programadas voltadas à educação em saúde bucal^8 , 9^ . Sugere-se que, nas edições futuras da PeNSE, sejam incorporadas questões relacionadas a essas dimensões.

A utilização de dados contextuais relativos às capitais e regiões (IDH e índice de Gini) com uma lacuna de cinco anos pode ter implicações para os resultados do estudo, pois refletem situações em diferentes pontos no tempo. Dados referentes ao ano de 2015 seriam mais adequados, mas não estavam disponíveis. Essa limitação é inerente aos estudos que utilizam dados secundários.

Outra limitação refere-se ao percentual de explicação resultante da análise CATPCA executada. Obtivemos um valor consideravelmente aceitável (acima da metade), porém seria desejável que ele estivesse mais próximo ao valor máximo. Da mesma forma, o coeficiente alfa de Cronbach total da referida análise traz um resultado bastante admissível, quando considerado o conjunto das três dimensões geradas^25^ . Contudo, isso não ocorreu quando as dimensões foram analisadas separadamente, e coeficientes abaixo de 0,7 foram encontrados. Apesar dessas limitações, os resultados indicam possível validade do indicador proposto, considerando a coerência e a plausibilidade verificadas. Ressalta-se que a análise CATPCA tem caráter exploratório, pressupondo a necessidade de outros estudos com o objetivo de testar a validade deste instrumento.

Concluímos que o indicador AEPSB foi associado a fatores contextuais das regiões, capitais e escolas, sendo mais elevado em regiões e capitais com maior desenvolvimento humano (IDH) e menor desigualdade socioeconômica (Gini) e em escolas da rede pública. Pesquisas adicionais devem ser realizadas para verificar essas associações em outros contextos.

Nossos achados sugerem a necessidade de ampliação das políticas públicas no Brasil buscando a melhoria dos ambientes escolares no que se refere aos aspectos políticos, curriculares, pedagógicos, estruturais e relacionais, que possam contribuir, direta ou indiretamente, para a promoção da saúde bucal. Ações institucionais estratégicas que considerem as diversidades socioeconômicas regionais são relevantes e necessárias para diminuir as iniquidades existentes.

O indicador AEPSB desenvolvido para a presente análise pode constituir uma ferramenta apropriada para avaliar o potencial de promoção de saúde bucal na escola a partir de variáveis categóricas relativas ao ambiente escolar, podendo ser adaptada a outros contextos e carecendo ainda de estudos adicionais de validação. A técnica de análise de dados empregada (CATPCA) para a construção do referido indicador pode ser replicada em outros estudos com objetivos semelhantes, podendo incluir variáveis adicionais para ampliar e favorecer as avaliações.
